# Studying technology use as social practice: the untapped potential of ethnography

**DOI:** 10.1186/1741-7015-9-45

**Published:** 2011-04-27

**Authors:** Trisha Greenhalgh, Deborah Swinglehurst

**Affiliations:** 1 Healthcare Innovation and Policy Unit, Centre for Health Sciences, Barts and The London School of Medicine and Dentistry, London E1 2AT, UK

## Abstract

Information and communications technologies (ICTs) in healthcare are often introduced with expectations of higher-quality, more efficient, and safer care. Many fail to meet these expectations. We argue here that the well-documented failures of ICTs in healthcare are partly attributable to the philosophical foundations of much health informatics research. Positivistic assumptions underpinning the design, implementation and evaluation of ICTs (in particular the notion that technology X has an impact which can be measured and reproduced in new settings), and the deterministic experimental and quasi-experimental study designs which follow from these assumptions, have inherent limitations when ICTs are part of complex social practices involving multiple human actors. We suggest that while experimental and quasi-experimental studies have an important place in health informatics research overall, ethnography is the preferred methodological approach for studying ICTs introduced into complex social systems. But for ethnographic approaches to be accepted and used to their full potential, many in the health informatics community will need to revisit their philosophical assumptions about what counts as research rigor.

## Background

'The existence of the experimental method makes us think we have the means of solving the problems which trouble us, but problem and method pass one another by.'

- Ludwig Wittgenstein, Philosophical Investigations, para 230 [1].

Health informatics - the study of information and communications technologies (ICTs) in healthcare - is a rapidly expanding field of research strongly influenced by (though extending beyond) doctors with an interest in computers. It emerged at around the same time as evidence-based medicine (EBM) and overlapped with the latter in several areas of work, notably the development of ICT systems to support large-scale epidemiological surveys and clinical trials; routinization of the use of Medline and other electronic databases; standardization of clinical practice via guidelines and automated decision support; and innovations such as computerized physician order entry (CPOE) aimed at reducing medical error [2-4].

Overall, the health informatics literature is hopeful and technophilic [5]. In this literature, ICTs are typically portrayed as potentially able to [a] incorporate (and thereby drive uptake of) evidence-based protocols and decision support; [b] overcome human failures and idiosyncracies; [c] ensure that clinical information is more complete, accurate and accessible; and [d] improve efficiency of healthcare transactions [6]. Health informatics is built largely though not exclusively on a positivist philosophy, determinist assumptions (that is, that a particular technology can cause a particular outcome) and experimental methodology. As Kaplan has put it:

'Traditionally, medical information systems evaluations have been conducted according to an experimental or clinical trials model of research. These evaluations focus on technical, economic, or other factors believed to affect systems' impacts. Some areas of systems evaluation are well-recognized in the medical informatics literature: (1) technical and systems features that affect systems use, (2) cost-benefit analysis, (3) user acceptance, and (4) patient outcomes. The factors believed to cause impacts were identified and the impacts measured. This kind of research design takes a variance approach; i.e., the focus of study is on how a variable changes as a result of some intervention, in this case, the information system.' [[7], page 95]

Controlled experimental and quasi-experimental studies oriented to determining the relationship between predefined variables such as completeness, accuracy, IT response times and morbidity (what Kaplan calls the variance approach) are commonly depicted as synonymous with robust health informatics research [8]. But these methodological approaches have been widely criticized in the social science literature for oversimplifying the social settings in which technologies are adopted and used (and also resisted and abandoned). Critics say they overlook issues such as meaning (is a computer a typewriter or a terminal?), power (who gets what access privileges and why?) and numerous other social and material influences on whether and how technologies are used (and whether they work) in particular contexts and settings leading to significant mismatches between the predicted and actual benefits of ICTs [9-11].

The limitations of experimental approaches to the social and organizational use of ICTs are beginning to be recognized within the health informatics discipline. Han *et al*, for example, set out to demonstrate in a large, quasi-experimental before-and-after study that mortality in a pediatric tertiary care center (dealing with very sick children, often transferred as emergencies from other centers) would be reduced by the introduction of a CPOE system to support 'safer' prescribing and dispensing of medication [12]. In fact, mortality increased significantly (from 2.80% to 6.57%) after the system was introduced. The authors, whose paper otherwise follows the experimental and quantitative style typical of biomedical papers, explained these unexpected findings thus:

'The usual chain of events that occurred when a patient was admitted through our transport system was altered after CPOE implementation. Before implementation of CPOE, after radio contact with the transport team, the ICU [intensive care unit] fellow was allowed to order critical medications/drips, which then were prepared by the bedside ICU nurse in anticipation of patient arrival. When needed, the ICU fellow could also make arrangements for the patient to receive an emergent diagnostic imaging study before coming into the ICU. A full set of admission orders could be written and ready before patient arrival. After CPOE implementation, order entry was not allowed until after the patient had physically arrived to the hospital and been fully registered into the system, leading to potential delays in new therapies and diagnostic testing (this policy later was rectified). The physical process of entering stabilization orders often required an average of ten clicks on the computer mouse per order, which translated to 1 to 2 minutes per single order as compared with a few seconds previously needed to place the same order by written form. Because the vast majority of computer terminals were linked to the hospital computer system via wireless signal, communication bandwidth was often exceeded during peak operational periods, which created additional delays between each click on the computer mouse. Sometimes the computer screen seemed frozen.' (page 1508-9)

This example offers some salient empirical and methodological lessons. Empirically, the commercial CPOE system (which had been extensively tested before release) did not perform as anticipated in real-world situations for three reasons. First, assumptions, constraints and access privileges which had been built into (or, to use the term preferred by sociologists, inscribed in) the technology as well-intentioned safety features could not be over-ridden to meet local contingencies, even when a child's life was at stake. Second, system designers missed critical elements of the collaborative work routine (input of key staff in a particular, time-dependent sequence) for emergency admission. Finally, electronic processes ran an order of magnitude more slowly than their written or spoken equivalent.

Methodologically, the above example shows that even relatively crude real-life observations presented in narrative form can convey much about the interaction between the material properties of technologies, time, place, space, and human action and interaction in the complex and fast-paced world of emergency healthcare. It suggests that richer insights could be generated by applying more sophisticated techniques of qualitative observation, for example, if detailed ethnographic field notes (what anthropologists call thick description [13]) were made; if these observational field notes were supplemented with video or screen-capture technologies; or if talk were recorded, transcribed and analyzed to facilitate study of the subtle complexities of interaction between humans and technologies.

Such methodological approaches could help health informatics researchers move beyond the determinist shackles of variance research and help them reconceptualize ICTs as what Harré has referred to as social substances, that is, in terms of their properties and meaning within a social world [14]. In this paper, we review how ethnography has been applied to study ICT use as social practice and propose that ethnographic approaches should be applied more widely in this field.

## Discussion

### What is ethnography?

The ethnographer immerses him or herself in a social situation and collects naturalistic data (that is, real-world observations rather than under experimental conditions) in a pragmatic, reflexive and emergent way [13,15]. Ethnographic data are rich in qualitative description (and sometimes also in visual imagery), allowing the researcher to interpret, to a greater or lesser extent depending on the degree of rigor applied (see below), what is really going on.

An important ethnographic tradition in the study of computers in the workplace is workplace studies, which emerged in the 1990s as part of a wider interdisciplinary field called computer-supported cooperative work (CSCW) [10,16-18]. Careful ethnographic observation in work settings showed that many work tasks which were previously assumed to be individual were actually collaborative. ICT design tends to focus on tasks performed by an individual user or on the relatively rare situation of focused collaboration on a single task. This deficiency may be particularly significant in healthcare where work typically comprises multiple, continuously multi-tasking individuals who come together for brief periods. The challenge is managing interdependencies between activities performed to achieve a goal, including handling conflicts of perspective [19]. Individuals must be aware both of the work of others and of the limitations of technologies, and make subtle and continuous adjustments to their own actions (articulation) to align with this.

Workplace studies drew on seminal theoretical work by ethnographer Lucy Suchman, who emphasized the limits of machine behavior compared to the situated (that is, tied to a particular situation in a particular context) interpretation of human actors. She rejected a key tenet of traditional human-computer interaction - that human action is individual, goal-oriented and based on rational plans - in favor of the notion that activity is collaborative and grows directly and organically out of the fine-grained particularities of a given situation [20]. She called for researchers to 'turn away from the experimental, the cognitive and the deterministic, to the naturalistic, the social and the contingent' [17].

The various research approaches which favor ethnography as a study design all share the view that ICTs cannot be meaningfully studied in isolation from the social situation in which they are used (or in which people decide not to use them), and all assume that technologies, in a sense, both shape and are shaped by human action. Technologies shape human action because they make some actions possible (for example, searching, aggregating), some impossible (for example by providing a limited set of options in a pull-down menu) and some unimaginable or socially difficult (for example by requiring the user to hit an emergency over-ride button). Technologies are shaped by human action because, for example, humans configure them, disable certain functionality, decide who may be trained to use them, and allocate differential access privileges to different people.

In relation to electronic patient records, for example, the notion of the record as a passive and neutral container for data about the patient is rejected in favor of a more nuanced, dynamic and active conceptualization of its role:

'The medical record is a tool...its does not 'represent' the work, but it feeds into it, it structures it in complex ways: it structures communication between healthcare personnel, shapes medical decision-making, and frames relations between personnel and patients.' [[21], page 297]

The ethnographer is less interested in assessing intrinsic features of technology (such as its data fields, coding structure or completeness or accuracy of the data it holds) and more interested in exploring ICT-supported social practices, that is, in the 'coordinated activities and performances which bring new situations into being but which are constrained by, in interaction with, and sometimes in tension with, surrounding practices and with what has gone before' [22]. Ethnography focuses on how technologies and the humans who are meant to use them actually perform under real, particular conditions of use (indeed, it has been described as a performative methodology).

Studying how technologies are used in social practice moves us on from studying either people or technologies (just as the study of drumming moves us on from studying either the drummer or the drum). Health informatics researchers sometimes talk in what Berg called 'essentialist' terms of a gap between reality (the lived body of the patient, or the practical reality of clinical medicine - messy, heterogeneous and impossible to code or classify) and a formal model-of-reality (the representation of this body and this practice in the electronic record - symbolic, clean, abstract and hence may be unproblematically coded and classified) [23]. Ethnographic methods, he suggested, allow us to go beyond lamenting this model-reality gap (an ultimately negative and technology-averse standpoint) and consider from a more positive perspective the ways in which skilful and creative human work is able to bridge this gap.

'More and more,...authors are calling for the need to reconfigure this dichotomous opposition between the formal and the informal. The positions are too entrenched; the rhetorics, too outdated; the foundations, too essentialist. Several authors have argued that formal tools can indeed transform workplaces in various ways but that this generative power can be attributed neither to the tool nor to the human workers. Rather, the generative power of this configuration lies in the interrelation of the formal with the informal. The distance between representation and represented, the existence of the gap, is here seen as the fruitful tension that can produce new worlds' [[23], page 406].

### Ethnographic research: philosophical foundations and quality criteria

Variables-centred (experimental and quasi-experimental) approaches and ethnographic approaches to the study of ICTs in healthcare have developed as distinct research traditions with remarkably little dialogue between them [5]. This is due in large part to differences in ontology (assumptions about the nature of reality), epistemology (how we can know that reality), methodology (what counts as robust study designs) and axiology (what is of value) [24].

For the positivist scientist (with whom most experimental ICT researchers would be happy to identify), there is a single reality which is knowable and probabilistic. Knowledge is seen as objective and dispassionate, and has a direct link to reality. The researcher is considered to be a detached observer of truth, and neither reflexivity nor relationship-building is given particular significance in the research process. Methodologically, the positivist researcher assumes a hierarchy of research designs, with quantitative experimental studies (for which the randomized controlled trial is the gold standard) seen as the most robust. The goal of positivist science is universal, transferable and predictive truth; hence models of reality achieved by statistical abstraction and generalization are valued very highly, and non-experimental approaches seen as necessarily less helpful [8].

Non-positivist research on ICTs span a range of philosophical positions, including interpretivist approaches such as sensemaking (which ask, for example, what meaning does this technology hold for different groups of actors in an organization? [25]), critical approaches (including feminist research on how technology may be used to further the interests of a dominant gender [26]) and recursive perspectives such as structuration theory and actor-network theory (which ask, for example, how micro-level phenomena such as the local understandings and actions of humans or the performance of technologies are shaped and constrained by wider influences and how, in turn, does micro-level action feed back into and change the wider socio-political context? [27,28]).

All these non-positivist traditions value immersion in uncontrolled real-world settings over conducting objective experiments. Such approaches are comfortable with multiple versions of reality. Indeed, ambiguity, paradox and conflict are viewed as valuable data and systematically analyzed for higher-order insights. Transferability of research findings is achieved not via statistical generalization (repeating the experiment or the observations across different settings) but via theoretical abstraction and generalization (that is, creating plausible and theoretically justifiable explanations, often based on the detailed study of the particular and the specific).

Ethnography is a very different kind of research from the controlled experiment. Rigorous ethnography is judged not in positivistic terms (for example how closely a predefined study protocol is adhered to, how tightly contextual variables are controlled, and so on) but in terms of three key interpretive criteria: authenticity (immersion in the case through extended fieldwork), plausibility (developing explanations of local phenomena which made sense to participants and drawing these together into a coherent overall narrative) and criticality (systematically questioning taken-for-granted assumptions, for example about who makes the decisions in a team) [29,30]. Whereas controlled experiments produce learning in terms of quantitative, predictive statements about the relationship between predefined variables, ethnographic studies produce a different kind of learning in terms of interpretive insights about actions and events placed in context [31].

### Some landmark ethnographic studies of ICT in healthcare

In a recent wide-ranging systematic literature review of electronic patient record research, we identified 12 purely ethnographic studies and a further 23 mixed-method studies which included an ethnographic element [5]. Some of these studies (those which we identified as rigorous according to the criteria above) are described below. This sparse sample contrasted with the 21 previous systematic reviews we identified which had been undertaken using Cochrane methodology and which covered more than 2,000 experimental and quasi-experimental studies on electronic patient records [5].

Drawing on Suchman's theoretical work (see above), Heath *et al *summarized a series of detailed ethnographic studies on what they called 'centres of coordination', data-dense and activity-rich areas where complex coordination of work was achieved by groups of people, such as air traffic control centers, financial trading centers and the nurses' desk in a busy emergency department [17]. Such centers typically relied on multiple sources of fast-changing information (paper, large electronic displays, digital print-outs, whiteboards, CCTV, verbal reports, and so on). A key finding from these studies was that there was no master overview but multiple diverse local perspectives, each constituted through the specific array of tasks, an ensemble of tools for performing those tasks, and the physical activity of the workers (including such subtleties as momentary glances at display screens).

Using a similar approach, Reddy *et al *studied a surgical intensive care unit in the USA [32]. They found that different professional groups (doctors, nurses and pharmacists) each had a different set of work practices which reflected the different focus, values and goals of their professions. The particular electronic record used on this unit was flexible and customizable, allowing different views for different professionals. Looking at these different screens allowed staff to see trends in changing variables and also orient themselves to what other professionals were doing, thus supporting the ordering and coordination of activity in a fast-changing clinical context. Importantly, the different screen views allowed both retrospective activity (aggregation of data to get a handle on the patient's progress over time done mainly by the physicians) and prospective activity (planning and coordinating care and procedures over the next few hours done mainly by nurses). Physical co-location (for example, several staff crowding round and discussing a particular screen on a shared computer) appeared essential for co-ordination of diverse work practices suggesting that problems may arise when ICTs are used to co-ordinate the work of geographically distributed staff. Placelessness may be technically achievable but it is a potential threat to patient safety.

Hartswood and Procter conducted a multi-site ethnographic case study of six breast screening centers in the UK, all of which used a particular ICT software package for registering and recalling patients and recording clinical findings [33]. They found that the complex work sequence of breast screening was a practical, situated accomplishment characterized by numerous workarounds and articulations, notably the use of handwritten notes on paper report forms, which served to augment the formally-recognized checks and performance audits. The authors comment: 'in practice, screening work is not so much organized to guarantee the flawless performance of each stage, but rather to support the safety and integrity of the overall process' (page 100).

Østerlund used a knowledge-in-action framework (in which knowledge was seen as something embodied and performed rather than merely possessed by individuals) to inform an 18-month ethnographic study of an emergency department in a US hospital and linked admission wards [34]. He showed how doctors and nurses use documents to organize their work practices that are distributed across teams. Members of staff recorded the same clinical data many times in different paper and electronic documents (a task he called 're-localization'). Each document served as a map and itinerary for a different constituency of people. The micro-detail of language use in medical records (in particular its indexicality, that is, the people and places implicitly or explicitly referred to in entries) provided a subtle but important structuring and ordering device for collaborative work [35,36]. Entries acquired new meaning when juxtaposed with other entries and/or re-entered by individuals with different roles.

Similarly, Ellingsen and Monteiro's ethnographic studies of electronic patient record systems in different departments in a Norwegian hospital [37,38] showed that seemingly redundant (repeated) or ambiguous (similar but not identical) entries served an important function: they created a space in which different teams could share information while maintaining different interpretations of it. They concluded that large, tightly integrated systems in which all data fields are rigidly standardized may be of less use in practice than smaller, more loosely coupled systems which make multiple, overlapping representations of knowledge possible [39,40].

## Summary

Whereas the dominant positivist paradigm in health informatics research tends to privilege the universal, the unified and the standardized (for example, the single, agreeable version of the electronic record in which each data item is entered only once and has a tightly-defined, non-negotiable meaning; common interoperability standards; shared protocols and guidelines, and so on), ethnographic studies have highlighted how collaborative work is achieved via multiple, iterative contributions to the emergent detail of particular situations. Each individual provides work fragments which acknowledge and respond to the input of others and to the here-and-now affordances and limitations of the technologies that are to hand. Collaborative healthcare work is thus not a formula to be followed or blueprint to be imposed but a vibrant, kaleidoscopic and unique patchwork quilt to be woven in real time from diverse inputs [37]. Inconsistencies and ambiguities between different staff perspectives (and different data fields and views in ICTs) provide scope for the local adjustments of emphasis and interpretation which help to achieve a more-or-less seamless experience for the patient. This was a critical missing element in the design of the CPOE system whose introduction was associated with an increase in mortality [12].

Philosophical differences between experimental and naturalistic approaches to health informatics research are profound and perhaps fundamentally incommensurable [41], though this incommensurability does not preclude useful mixed-method studies [42]. In recent years, qualitative methods in general and ethnography in particular have become more popular in the healthcare community [15]. The research which informed this paper, for example, was funded by a new methodologies call from the UK Medical Research Council http://www.mrc.ac.uk. Ironically, ethnography as a method is as old as the discipline of anthropology. What makes it new is its application to traditionally positivist fields of inquiry (where deeply-held paradigmatic assumptions threaten to limit its application and credibility) and its application to health informatics.

We suggest that it is time for research sponsors, researchers, journal editors, trainers and practitioners to move beyond the assumption that whatever the research question, a large, controlled, technology-on-versus-technology-off experiment will necessarily provide better evidence than a small-scale, carefully conducted ethnographic case study. Where appropriate, we need to commission such studies, ground them theoretically, conduct them rigorously, review them critically, learn from them, build on them and take account of their insights when designing new systems. None of these things is currently happening to the extent that is needed, which is why health informatics research remains dominated by large-scale studies that privilege method over theory and abstraction over illumination.

To illustrate this point, the most recently published recommendations for a health informatics training syllabus for professionals at bachelor, masters and doctorate level refers to socio-technical issues and qualitative research once each (the former unelaborated and the latter in relation to triangulation against quantitative research); the extensive and detailed syllabus, which references 90 key texts, makes no mention of either ethnographic or socio-technical co-design methods [43]. These study designs (and the epistemological assumptions on which they are based) appear to have been deemed out of scope.

As the studies described in this article show, ethnography has much to offer health informatics research, but its contribution may remain in the shadows until the field acknowledges the need not merely for new methodologies but also new ontologies, new epistemologies and new definitions of what is of value. We offer this paper as a contribution to the urgent debate which we believe is needed on ways of knowing in eHealth research.

## Abbreviations

CCTV: Closed-circuit television; CPOE: Computerized physician order entry; CSCW: Computer-supported cooperative work; EBM: Evidence-based medicine; ICT: Information and communications technology; ICU: Intensive care unit; USA: United States of America;

## Competing interests

The authors declare that they have no competing interests.

## Authors' contributions

TG conceptualized the paper with input from DS. DS is conducting a PhD study based on ethnographic methods which inspired the ideas in this paper. TG and DS wrote the paper. All authors read and approved the final manuscript.

## Pre-publication history

The pre-publication history for this paper can be accessed here:

http://www.biomedcentral.com/1741-7015/9/45/prepub
